# Masseter Muscle in Somatosensory Tinnitus and Potential Therapeutic Role for Botulinum Toxin Type A: A Scoping Review

**DOI:** 10.3390/toxins18070310

**Published:** 2026-07-17

**Authors:** Jacopo Gardellin, Marta D’Angelo, Lorenzo Spadotto, Giacomo Checchin, Giovanni Gaetti, Luca Gambolò, Giuseppe Stirparo, Nicola Merli

**Affiliations:** 1 Medical Art, Piazza della Repubblica 5, 20121 Milan, Italy; 2 SIMED—Società Italiana di Medicina e Divulgazione Scientifica, Via Trento 42, 43122 Parma, Italy; 3 Synolon Medicina Estetica, Via Rialto 16, 30020 Noventa di Piave, Italy; 4 Department of Neuroscience and Rehabilitation, University of Ferrara, 44121 Ferrara, Italy

**Keywords:** tinnitus, masseter muscle hypertonia, masseter muscle hypertrophy, bruxism, temporomandibular disorder, botulinum toxin

## Abstract

**Background**: Increasing evidence supports the existence of a somatosensory subtype of tinnitus, in which craniocervical–craniomandibular structures can modulate tinnitus perception also through central auditory pathways. Despite growing mechanistic evidence, no targeted pharmacological intervention has been validated for this subtype. **Objectives**: This study aimed to synthesize the anatomical and neurophysiological rationale linking masseter hyperactivity to somatosensory tinnitus and to discuss BoNT/A as a hypothesis-generating therapeutic option requiring clinical validation. **Methods**: A PRISMA-ScR-guided scoping review was conducted in MEDLINE and EMBASE, using three complementary search lines (last search: 31 May 2026). **Results**: Of 284 records screened, four studies met eligibility criteria: three from the search strategy on BoNT/A and tinnitus and one from the search strategy on tinnitus and masticatory dysfunction. One additional record (Ranoux and Levine, 2024) was identified through manual search, describing tinnitus improvement following periauricular and splenius capitis BoNT/A injections, with masseter/temporalis injection proposed as an alternative site in the same protocol. **Conclusions**: Current anatomical and neurophysiological evidence supports a plausible link between masseter hyperactivity and somatosensory tinnitus in selected patients. BoNT/A is established for reducing masseter hyperactivity and may theoretically reduce abnormal somatosensory input; however, direct clinical evidence for tinnitus improvement remains insufficient. Its use should be considered experimental or hypothesis-based, within a multidisciplinary framework and with standardized outcome assessment. Prospective controlled trials are needed.

## 1. Introduction

Tinnitus, especially when persistent and bothersome, can severely affect quality of life, causing sleep disturbance, difficulty in concentration, social and emotional impairment, anxiety, and depression [1,2,3,4]. Among tinnitus subtypes [5,6,7], somatosensory tinnitus, in which non-auditory inputs modulate perception, has attracted growing interest, particularly in relation to craniomandibular dysfunction, bruxism, and masseter muscle pathology.

Botulinum toxin type A (BoNT/A) is established for reducing hyperactivity in masticatory muscles across aesthetic and functional indications, including bruxism and temporomandibular disorders (TMDs) [8,9], but direct clinical evidence supporting its use specifically for tinnitus remains limited [10,11,12]. The neurophysiological basis for this connection hinges on the dorsal cochlear nucleus (DCN), where trigeminal somatosensory inputs from the masseter converge with auditory pathways, providing a plausible substrate for peripherally driven tinnitus modulation.

Given the absence of sufficient homogeneous primary studies and direct evidence in support of a meta-analytic approach, a scoping review design was selected to map the available evidence, identify knowledge gaps, and generate hypotheses for future controlled trials—specifically regarding the anatomical and neurophysiological rationale linking masseter hyperactivity to somatosensory tinnitus and the potential role of BoNT/A as a hypothesis-driven therapeutic option.

## 2. Results

After applying the search strategy and study selection criteria, and after screening for title, abstract and full-text assessment, the first search line produced three eligible studies, while the second identified one study eligible for inclusion [13,14,15,16]. Considering the limited evidence available, one additional record was identified outside the primary database search: a conference abstract by Ranoux and Levine [17] describing two patients with non-pulsatile tinnitus who experienced symptom improvement following botulinum toxin injections into the periauricular muscles and splenius capitis.

**Figure 1:**
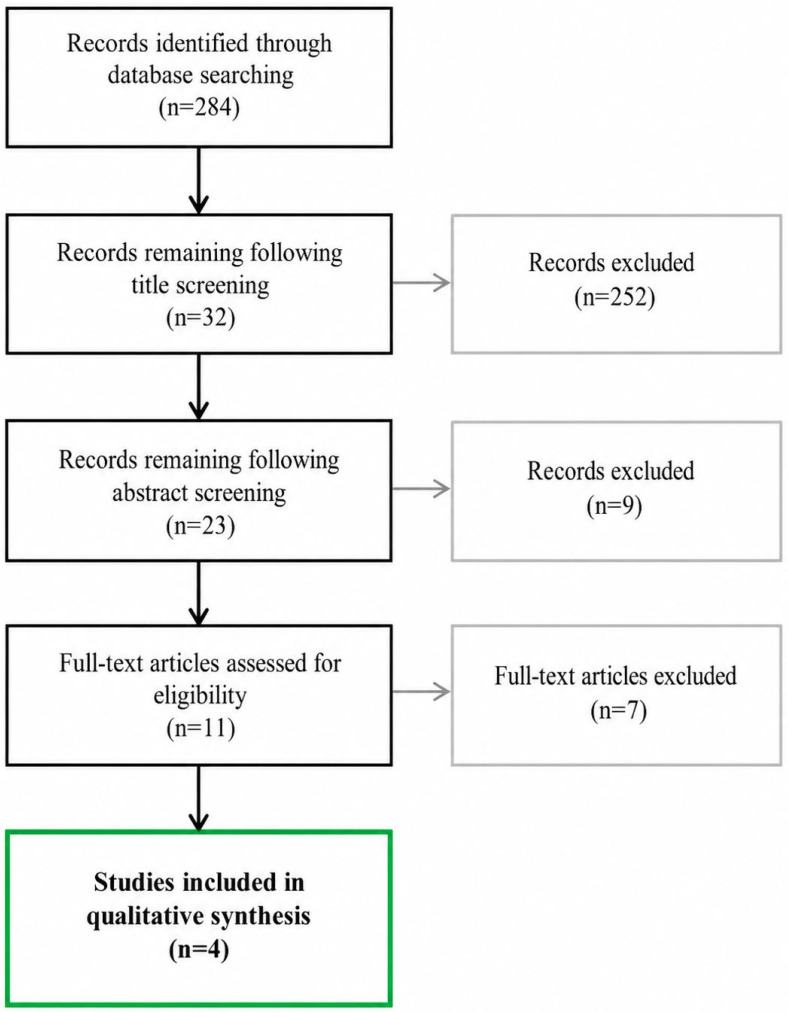
PRISMA-ScR flow diagram illustrating the research strategy.

A PRISMA-ScR flow diagram illustrating the study selection and screening process is provided in Figure 1.

Table 1 provides a structured overview of the included studies, detailing study design, population, intervention, outcome measures, and main findings.

## 3. Discussion

### 3.1. Tinnitus and Its Somatosensory Subtype

Tinnitus is defined as the perception of sound in the absence of an external auditory stimulus [5,6,7,18,19,20]. A recent systematic review and meta-analysis estimated that the overall global prevalence of any type of tinnitus among adults is 14.4%. Despite extensive research, the underlying aetiology remains unknown in a significant percentage of patients (primary or idiopathic tinnitus) [5,6,18,19,20,21,22].

Somatosensory or somatic tinnitus (ST) is characterized by modulation of tinnitus by movements or pressure involving the head, neck or jaw, as well as coexisting musculoskeletal disorders [23]. ST is defined by Haider et al. [24] as “a generally agreed subtype of tinnitus that is associated with activation of the somatosensory, somatomotor, and visual-motor systems. A key characteristic of somatosensory tinnitus is that is modulated by physical contact or movement”. Within the broader heterogeneous population, patients with ST may represent a subgroup in which targeted neuromuscular interventions, such as therapies directed at the craniocervical and craniomandibular systems, may be more effective than purely auditory approaches [13,25,26]. Ralli [27] reported that this subtype may be present in approximately 33% and 85% of patients with tinnitus.

Classifications based on duration further define tinnitus as acute (present for less than 3 months), subacute (3–6 months) and chronic (more than 6 months) [5,6,7,21]. Tinnitus can be classified as subjective or objective and pulsatile or non-pulsatile, and the condition may be constant or intermittent and unilateral or bilateral.

In cases of suspected somatosensory involvement, recent international guidelines have proposed an approach that integrates both the patient’s medical history and focused examinations. Further evaluation by dental specialists and ENT doctors may be necessary to assess masseter muscle hyperactivity and cervical pathology [28,29,30].

### 3.2. The Masseter Muscle as a Pathophysiological Hub

The masseter is one of the primary muscles of mastication; it consists of superficial and deep parts. Its main function is to elevate the jaw and it is innervated by the mandibular branch of the trigeminal nerve (motor branch), closely connected to the tensor tympani and temporomandibular joint (TMJ) [31,32,33,34,35,36,37]. In addition to the masseter, several other muscles (temporalis [38], pterygoids [39,40,41,42,43], sternocleidomastoid [44,45], trapezius [45], and tensor tympani [46]) within the cervical and craniomandibular region play important roles in jaw movement and have the potential to influence tinnitus symptoms. The interaction between masticatory muscles and the ear was termed otognatic syndrome by Myrhaug (1964) [47] and otomandibular syndrome by Bernstein (1969) and Arlen (1977) [46]. From a neuroanatomical standpoint, the primary proprioceptive neurons of the masseter are unique in that their cell bodies are located not in a peripheral ganglion but within the brainstem itself. Unlike the spindle afferents of spinal muscles (whose cell bodies reside in peripheral dorsal root ganglia), the primary proprioceptive neurons of the masseter project to the mesencephalic trigeminal nucleus (MTN), the only sensory nucleus of the central nervous system to house first-order proprioceptive neuron cell bodies. This unique arrangement, recently characterized at the molecular and developmental levels [48], positions the MTN as a direct interface between masseter-derived sensory input and central trigeminal circuits, and carries plausible specific implications for the pharmacological modulation of masseter proprioception discussed in Section 3.5.1.

An increasing number of studies have consistently described that not every case of tinnitus originates exclusively from the auditory pathways; the musculoskeletal system (particularly TMD and dysfunction of the cervical spine) is responsible for a significant percentage of cases of somatosensory tinnitus [5,25,49,50].

In 1993, Jastreboff and Hazell [51] shifted the understanding of tinnitus by proposing a neurophysiological model focused on central mechanisms. Their framework suggests tinnitus results from changes in neural processing within central auditory pathways and their links to other brain systems. The auditory and the somatosensory systems converge and interact within the DCN. If the activity of the DCN’s somatosensory interacting fusiform cells exceeds an individual’s tinnitus threshold, then tinnitus results [52]. As reported by several authors, an increased spontaneous firing rate of neurons in the central auditory system is one possibility for the neural substrate of tinnitus [53,54,55,56]. Salvinelli and colleagues [57] proposed the “double-hit” hypothesis, where a neural signal in auditory or somatosensory pathways causes higher auditory centres to mimic activity patterns seen with external sounds. They noted that TMJ is innervated by the auriculotemporal nerve (ATN), a branch of the mandibular trigeminal nerve. Due to anatomical proximity, forced contralateral TMJ movements may irritate the ATN, activating neural responses relayed to the DCN. Somatosensory signals via the ATN could thus induce abnormal neural activity in central auditory pathways.

Salvinelli et al. [57] further proposed that reduced serotonergic modulation in the dorsal cochlear nucleus may lower the threshold for abnormal neural activity, potentially reinforcing tinnitus memory circuits (a mechanism that may interact with the somatosensory inputs described above).

Moreover, evidence indicates that cervical spine issues, especially in the upper segments (C1–C3) and craniomandibular system, can directly influence or modulate somatosensory tinnitus through neural connections with auditory pathways [58].

Clinical evidence supports the relevance of masseter hyperactivity specifically. In a sample of 163 patients, jaw muscle maneuvers (particularly teeth clenching and jaw opening) were associated with increased tinnitus loudness in a significant proportion of cases, corroborating the clinical relevance of masticatory muscle activity in tinnitus modulation [59].

Somatic modulation of tinnitus demonstrates multisensory integration, where one sense influences another. Numerous studies show that many people can alter their tinnitus by moving their head, neck, or limbs [59,60]. D’Amato et al. have recently reported that patients with somatosensory tinnitus exhibit significantly higher frequencies of oral behaviours [61]. Recent electrophysiological evidence strengthens this connection. In a 2025 study, Didier et al. compared masticatory muscle activity in patients with TMD with and without somatosensory tinnitus, demonstrating that muscle activity patterns differ significantly between groups, suggesting that altered masseter and temporalis function may contribute to tinnitus modulation [30].

Muscle tone and force output rise during parafunctional activities, which include tooth grinding, clenching, chattering, nail biting, tongue pressing, and frequent gum chewing, potentially resulting in intra-articular soft tissue damage [62,63]. These interactions among muscle, bones and cartilage create complex pathways involving growth factors and interleukins, which are still not completely understood [64].

### 3.3. Clinical Relevance and Diagnosis

The pathophysiological mechanisms described above (trigeminal–auditory convergence, parafunctional overloading, and myofascial sensitization) frequently occur in the context of overlapping clinical entities that require explicit differentiation (Table 2).

Clinical criteria for suspicion of somatosensory tinnitus include tinnitus arising or worsening with bruxism, TMJ pain, or recent head/neck trauma (i); intensity or pitch changes with movements of the jaw, neck, or even eyes (ii); and relief after physical therapy, muscle relaxation, or dental bite adjustment (iii). Recommendations for diagnostic assessment through clinical history and clinical examination are generally founded more on expert consensus than on evidence derived from systematic controlled trials.

### 3.4. Treatment and Management

A comprehensive diagnostic work-up is a prerequisite [70,71]. European guidelines [21], NICE recommendations [72], and recent expert position statements guide the management of tinnitus. Clinical practice guidelines for TMD aim to guide decision-making but often differ in recommendations and have notable development limitations. Four of the five latest guidelines lack methodological details and only list treatment options without addressing comparative effectiveness or evidence certainty [68].

Tinnitus counselling is the first step, providing information, coping strategies, stress management, relaxation techniques, emotional support, and boosting self-efficacy. Auditory treatments (hearing aids and sound therapy) are strongly recommended in the presence of significant hearing loss. Evidence-based guidelines grant a strong recommendation to Cognitive Behavioural Therapy (CBT) for reducing tinnitus-related distress and improving quality of life. CBT alters maladaptive cognitive and behavioural responses, such as catastrophic misinterpretation and safety-seeking avoidance [21,22,60].

Pharmacological treatments (e.g., muscle relaxants, anticonvulsants, and antidepressants) have limited effectiveness for primary tinnitus but are considered for relevant comorbidities [72,73,74,75,76,77,78,79]. Current guidelines recommend against the routine use of anticonvulsants or antidepressants for primary tinnitus treatment.

Orofacial physical therapy, which includes manual mobilization, massage of the masticatory muscles, and stretching exercises, has demonstrated significant efficacy [80,81]. Van der Wal et al. [81] and De la Serna et al. [82] observed a clinically significant improvement after orofacial therapy.

Conversely, while occlusal splints are standard for TMD, their isolated effectiveness for tinnitus improvement remains mixed, often requiring combination with other therapies to be effective [66,80,83,84].

Targeted deactivation of myofascial trigger points (MTPs) in the masseter represents a further avenue: MTPs are strongly associated with tinnitus modulation and lateralization, and their palpation frequently produces transient tinnitus alteration—implicating both somatosensory–auditory interactions and the sympathetic nervous system [25,66]. Dry needling has shown efficacy in reducing emotional and functional discomfort associated with chronic somatosensory tinnitus [84,85,86,87]. Other neuromodulation and psychological approaches (e.g., photobiomodulation, neuromodulation, eye movement desensitization and reprocessing) have been explored but are beyond the scope of this review.

### 3.5. Botulinum Toxin Type A: Mechanistic Rationale and Available Evidence

#### 3.5.1. Mechanisms of Action Relevant to Masseter Muscle and Pain

BoNT/A is a neurotoxin with seven serotypes, and due to its well-established safety profile and efficacy, BoNT/A is the most thoroughly researched and widely implemented in clinical practice (aesthetic medicine, oncology, neurology, rehabilitation, and dentistry). By inhibiting acetylcholine release at the neuromuscular junction, BoNT/A reduces excessive muscle activity and pathologic somatosensory input from the masseter [65]. In vitro and in vivo studies have demonstrated that BoNT/A inhibits the release of nociceptive mediators such as glutamate, substance P, and calcitonin gene-related peptide (CGRP) from nociceptive fibres [88,89], suggesting a direct anti-nociceptive action. Moreover, through a peripheral mechanism, BoNT/A has also been shown to inhibit central sensitization of central trigemino-vascular neurons, a key process in migraine [90].

Beyond its well-known action on extrafusal muscle fibres, BoNT/A also acts on muscle spindles by blocking the γ-motoneurons that regulate spindle sensitivity. This reduces afferent sensory output from the muscle independently of its effect on contraction strength [91,92]. In the masseter specifically, animal studies have demonstrated a reduction in spindle afferent discharge following BoNT/A injection while muscle tension remained unaffected [93]—a finding whose functional significance is amplified by the unique neuroanatomy of masseter proprioception: spindle afferents project directly to the MTN (see Section 3.2), placing BoNT/A-induced proprioceptive modulation within central trigeminal circuits from the first synaptic relay. The broader relevance of spindle-mediated central effects is supported by neuroimaging evidence in humans: a single BoNT/A injection into dystonic neck muscles produced widespread normalization of sensorimotor cortical activation in non-injected body segments, interpreted as correction of maladaptive plasticity through reduced aberrant spindle afferent input [94,95]. Whether an analogous proprioceptive mechanism in the masseter could contribute to the modulation of the trigeminal–DCN pathway relevant to somatosensory tinnitus has not been investigated and remains speculative.

Recent longitudinal studies using myotonometry have quantified these effects, showing that BoNT/A produces measurable, long-lasting reductions in masseter muscle tension and stiffness [65,96]. Electromyographic studies have demonstrated a reduction in neuromuscular activity after BoNT/A treatment.

#### 3.5.2. BoNT/A Administration: Dose and Accuracy

Reported BoNT/A doses for masseter injection in the TMD and bruxism literature typically range from 24 to 60 U per side (onabotulinumtoxinA/incobotulinumtoxinA equivalent) or 60–300 U for abobotulinumtoxinA, administered intramuscularly at three to five points within the muscle belly, with effects generally lasting 3–6 months [97]. Higher cumulative doses have been reported in Asian populations with more pronounced masseter prominence, where anatomical differences in masseter development and mandibular angle morphology may justify dose adjustment [98].

Precision is a key factor for the correct administration of BoNT/A; for this reason, ultrasound (US) guidance has increasingly been adopted to improve the injection accuracy of BoNT/A into facial and masticatory muscles, allowing real-time visualization of muscle boundaries, vascular structures, and the parotid gland, thereby reducing the risk of diffusion into adjacent tissue [99,100,101,102]. While US-guided injection protocols for the masseter are well described in the context of masseter hypertrophy and bruxism [103], their application specifically for tinnitus-related indications has not yet been investigated and represents an area for future methodological standardization [104].

#### 3.5.3. Indirect Evidence: BoNT/A in Temporomandibular and Masticatory Muscle Conditions

Contemporary clinical practice guidelines and recent studies regarding temporomandibular disorders primarily advocate for conservative, reversible interventions in the management of chronic TMD pain. Although guidelines explicitly recognize somatosensory influences, they do not yet provide detailed, mechanism-based recommendations for pharmacologic or minimally invasive treatments targeting the musculoskeletal system, with limited or no routine endorsement of BoNT/A [68].

At the same time, guideline panels and expert consensus statements highlight the importance of developing personalized treatment strategies and call for research in under-represented subgroups, including patients with somatosensory tinnitus [22,26]. From a clinical perspective, these patients often experience substantial burden and may have exhausted standard auditory-based therapies [13]. In this setting, interventions directed at masseter hypertonia [65] represent a pathophysiologically grounded, though still experimental and hypothesis-based, treatment option that directly addresses a plausible peripheral driver of tinnitus.

#### 3.5.4. Direct Evidence on BoNT/A in Tinnitus

A few reports have described tinnitus improvement with different sites and techniques of BoNT/A administration. Direct clinical evidence on BoNT/A specifically for tinnitus remains limited to a small number of studies, most of which address subtypes other than the somatosensory phenotype (including palatal tremor, hemifacial spasm, and middle ear myoclonus) or report periauricular injection sites rather than masseter-targeted approaches.

Láinez and Piera [14] in their narrative chapter described the potential benefit of BoNT/A treatment in tinnitus patients, underscoring the neurophysiological link consistent with trigeminal–auditory modulation (i), the nociceptive actions of BoNT/A (ii), and its peripheral mechanism (iii).

Stidham et al. [15] evaluated BoNT/A in a small sample of tinnitus patients receiving either BoNT/A or placebo at different times and observed a positive effect after subcutaneous periauricular injections of onaBoNT/A. The authors recommend a larger study to document the generalizability and repeatability of the results before drawing definitive conclusions.

Ranoux and Levine (2021) [16], studying a cohort of 57 patients with chronic migraine variably treated with onaBoNT/A injections in the temporalis, corrugator, and trapezius muscles, identified five subjects with demonstrated chronic tinnitus improvement. Specifically, tinnitus completely disappeared in two patients, whilst in the other three an estimated loudness reduction by more than 70% was observed.

Ranoux and Levine (2024) [17], in their two-patient report published as a conference abstract, observed non-pulsatile tinnitus improvement following injections targeting periauricular muscles and splenius capitis—proposing the following approach to treating non-pulsatile tinnitus using incoBoNT/A: injection of 10 U to each periauricular muscle, 15 U in splenius capitis, or 15 U in the temporalis and masseter muscles.

Collectively, these studies are insufficient to establish clinical efficacy: sample sizes are small and methodologies heterogeneous, and none specifically enrolled patients with somatosensory tinnitus related to masseter hyperactivity.

### 3.6. Proposed Clinical Framework

The anatomical and pathophysiological evidence presented supports the masseter muscle as a plausible peripheral driver of somatosensory tinnitus, particularly through its trigeminal connections to the dorsal cochlear nucleus and its role in parafunctional loading of the TMJ. This rationale, however, remains inferential: it is based on neuroanatomical plausibility and indirect clinical observations rather than prospective interventional data.

Within this framework, a clinically coherent approach can be evaluated for a highly selected population: patients in whom somatosensory tinnitus is plausibly related to masseter hyperactivity, after thorough exclusion of audiological and neurological causes, and in whom standard conservative reversible therapies have proven insufficient. In this subgroup, BoNT/A targeting the masseter could be explored in future controlled studies, within a multidisciplinary setting, as a hypothesis-driven intervention.

This position is consistent with the broader trajectory of guideline development in both TMD and tinnitus management, which increasingly calls for personalized treatment strategies and targeted research in under-represented subgroups. It does not constitute a treatment recommendation [26]. As reported by Delcanho et al., due to the considerable variations in study methods and results, small sample size, and absence of an established injection protocol, the role of BoNT/A needs further and more detailed research [10].

## 4. Limitations of This Study

First, this study was conducted as a scoping review rather than a systematic review, and the literature selection was not based on a predefined protocol with structured risk-of-bias assessment. The included studies originate from different countries and clinical settings, with heterogeneous patient populations, diagnostic criteria for somatosensory tinnitus, and varying definitions of masseter hypertonia and temporomandibular disorders. Second, most of the available clinical evidence (particularly regarding botulinum toxin type A in tinnitus) derives from the low quality of evidence studies. Third, outcome measures across studies are not uniform, ranging from subjective tinnitus questionnaires to electrophysiological or biomechanical assessments, making direct comparison difficult. Finally, the pathophysiological link between masseter hyperactivity and tinnitus remains largely inferential and based on neuroanatomical and neurophysiological plausibility rather than robust prospective interventional trials. Additionally, the search was restricted to English-language publications, which may introduce selection bias by excluding potentially relevant studies published in other languages. These limitations highlight that future research should focus on evaluating current evidence-based treatments for TMD, MMH, MH, and bruxism, as well as the correlation with somatosensory tinnitus. Furthermore, well-designed, adequately powered, prospective studies with standardized diagnostic criteria and validated outcome measures are needed to address this coexisting condition.

## 5. Conclusions

Current anatomical and neurophysiological evidence supports a plausible link between masseter hyperactivity and somatosensory tinnitus in selected patients, including through proprioceptive pathways whose modulation by BoNT/A has been documented in analogous craniocervical contexts. BoNT/A is established for reducing masseter hyperactivity and may theoretically reduce abnormal somatosensory input; however, direct clinical evidence for tinnitus improvement remains insufficient. Its use should be considered experimental or hypothesis-based, within a multidisciplinary framework and with standardized outcome assessment. Future prospective trials should address the key methodological gaps identified by this review: validated diagnostic criteria for somatosensory tinnitus as inclusion criteria, injection protocols targeting masticatory or cervical muscles, and adequate follow-up with a control arm. Prospective controlled trials addressing these elements are the necessary next step to determine whether this rationale translates into clinical benefit.

## 6. Materials and Methods

The present scoping review was not designed to test a priori hypotheses; rather, it aims to map the available evidence and identify gaps in the current literature. Studies of patients with tinnitus, specifically somatosensory tinnitus, occurring in association with temporomandibular disorders (TMDs), bruxism, masseter hypertrophy, or masticatory muscle dysfunction were considered. The use of botulinum toxin injections targeting the masseter muscle and/or other masticatory muscles for the management of tinnitus or tinnitus-related conditions, in any clinical setting (including otolaryngology, audiology, dentistry, oral and maxillofacial surgery, pain medicine, neurology, and rehabilitation) was considered. No protocol registration was completed prior to the conduct of this review; this is acknowledged as a limitation and is further discussed in Section 5.

Given the limited direct evidence on BoNT/A injections into the masseter muscle specifically for somatosensory tinnitus, and to maximize sensitivity, a multi-step search strategy was conducted across MEDLINE and EMBASE databases from inception to 31 May 2026. When the three search lines were combined into a single query combining all three domains (BoNT/A, masticatory muscles, and tinnitus), limited results were retrieved after applying study-type filters, confirming the near-total absence of direct clinical evidence and justifying a multi-step approach.

The search strategy consisted of three complementary lines, detailed in Table 3, covering direct evidence (BoNT/A and tinnitus), pathophysiological rationale (tinnitus and masticatory dysfunction), and contextualisation (BoNT/A and masticatory muscle conditions).

Studies exclusively investigating botulinum toxin for masseter hypertrophy, bruxism, or temporomandibular disorders without reporting tinnitus or tinnitus-related outcomes were excluded during screening.

Inclusion criteria encompassed systematic reviews, meta-analyses, randomized controlled trials, clinical trials, observational studies, and scoping reviews published in English. Studies focusing exclusively on objective tinnitus subtypes without somatosensory features (e.g., palatal tremor or middle ear myoclonus) were excluded.

The overall reference list was further broadened with additional sources identified through citation tracking and manual search, selected for their relevance to the anatomical, neurophysiological, or clinical aspects discussed.

Titles and abstracts were screened independently by two reviewers (L.S. and N.M.), with full-text assessment performed for all potentially eligible records. Disagreements were resolved by consensus. Data were subsequently extracted independently by the same two reviewers using a standardized charting template.

A critical risk-of-bias assessment was not performed because the direct evidence was limited and heterogeneous, and the review primarily aimed to map the existing literature and identify current knowledge gaps.

## Figures and Tables

**Table 1:** Charting of included studies.

Study (Ref.)	Design	Population	Search Line	Intervention	Outcome Measure	Main Finding
Michiels, 2023 [13]	NR	ST patients; broad population	2—Pathophysiological rationale	NA	Narrative synthesis of diagnosis and treatment options	Identifies neck/jaw region dysfunction as a key contributor to ST; CN neuron interaction in ST; ST treatment with musculoskeletal treatment and other techniques, such as BoNT/A
Láinez & Piera, 2007 [14]	NR	ST patients;	1—Direct evidence	NA	Narrative synthesis of treatment options with BoNT/A focus	TMJ disorders are often associated with tinnitus; BoNT/A actions could explain tinnitus relief
Stidham et al., 2005 [15]	DB-RCT (crossover)	Tinnitus patients; n = 26; control group receiving placebo	1—Direct evidence	BoNT/A injection into periauricular muscles (not masseter-targeted)	Tinnitus loudness and subjective distress	Results suggest a positive effect of BoNT/A on tinnitus (recommendation for a larger study to document the potential benefits)
Ranoux & Levine, 2021 [16]	PCS′	CM patients; n = 57; of these, n = 5 with tinnitus	1—Direct evidence	BoNT/A injection into the temporalis, corrugator, and trapezius, depending on the patients’ symptoms	In 2 patients, tinnitus disappeared, and in 3 patients, it was reduced by more than 70%	Tinnitus improvement reported
Ranoux & Levine, 2024 [17]	CA*	CM patients with non-pulsatile tinnitus; n = 2	1—Direct evidence	BoNT/A injection into the splenius and periauricular muscles	1 patient with no tinnitus for 4 months 1 patient with no tinnitus for 2 years	Proposition of a therapeutic approach for non-pulsatile tinnitus

NR = Narrative Review. DB RCT = Double-Blinded Randomized Clinical Trial. PCS′ = Prospective Cohort Study with serendipitous finding. CA* = conference abstract, not a full peer-reviewed article. ST = somatosensory tinnitus. CN = cochlear nucleus. TMJ = temporomandibular joint. CM = chronic migraine. Note: Ref. [17] was identified through manual search outside the formal database screening process and is not counted among the four studies in the PRISMA-ScR flow diagram. It is included here for completeness given its relevance to periauricular/splenius-targeted BoNT/A in tinnitus, with the masseter proposed as an alternative site.

**Table 2:** Different clinical entities involved in the pathophysiological rationale.

Clinical Entities	Definition	Notes
Masseter Hypertrophy (MMH) [28]	An enlargement of the masseter muscle, presenting as a visible or palpable swelling at the mandibular angle.	Idiopathic or associated with increased functional loading. Pain could be absent.
Masseter Hypertonia (MH) [65]	Persistent increase in resting muscle tone and excessive activity during function.	Frequently linked to parafunctional behaviours.
Myofascial Pain (MP) [66]	A pain syndrome arising from hyperirritable myofascial trigger points within skeletal muscle or its fascia.	Pain patterns can include the ear and otologic symptoms.
Bruxism [67]	A repetitive masticatory activity characterized by clenching or grinding the teeth and/or thrusting the jaw.	Behavioural phenomenon. May occur awake or when asleep. Can cause tooth wear, TMJ and/or muscular pain, joint locking and noise. May act as a risk factor for TMD and MH.
Temporomandibular Disorder (TMD) [68,69]	A heterogeneous group of conditions of musculoskeletal and neuromuscular conditions affecting the temporomandibular joint (TMJ), masticatory muscles, and associated structures.	Can cause pain in the jaw, face, and neck, and may present with headaches, earache, clicking, popping, or crepitus in the temporomandibular joint, and impaired mandibular function.

**Table 3:** Search strategy per database and search line.

Search Line	Focus	Database	Boolean Strings	Results
1—Direct evidence	BoNT/A and tinnitus	MEDLINE (PubMed), EMBASE	(“botulinum toxin” [tiab] OR Botox [tiab] OR BoNT/A [tiab]) AND (tinnitus [tiab] OR “somatosensory tinnitus” [tiab])	3 eligible studies
2—Pathophysiological rationale	Tinnitus and TMD	MEDLINE (PubMed), EMBASE	(tinnitus [tiab] OR “somatosensory tinnitus” [tiab]) AND (masseter [tiab] OR bruxism [tiab] OR “masseter hypertrophy” [tiab] OR “masseter hypertonia” [tiab] OR “myofascial pain” [tiab] OR “temporomandibular disorder *” [tiab] OR TMD [tiab])	1 eligible study
3—Context only *	BoNT/A and TMD	MEDLINE (PubMed), EMBASE	(“botulinum toxin” [tiab] OR Botox [tiab] OR BoNT/A [tiab]) AND (masseter [tiab] OR “masseter hypertrophy” [tiab] OR bruxism [tiab] OR “temporomandibular disorder *” [tiab] OR TMD [tiab])	NA

* Search Line 3 was implemented exclusively to contextualize existing evidence on BoNT/A in masticatory muscle conditions and did not contribute to the primary selection of included studies. tiab = title/abstract field tag.

## Data Availability

No new data were created or analyzed in this study. Data sharing is not applicable to this article.
